# Mr. Christie's Observations on Gun-Shot Wounds

**Published:** 1799-12

**Authors:** J. Christie

**Affiliations:** 27th Foot. Schagen, North Holland


### *To the Editors of the Medical and Phyſical Journal.*

GENTLEMEN,

AFTER the embarkation of the continental expedition, under the orders of His Excellency, Sir RALPH ABERCROMBIE, the command of which has ſince been aſſumed by His Royal Highneſs the Duke of YORK, I conceived the thought of ſending occaſionally, for inſertion in your extenſive Miſcellany, ſuch caſes of the gun-ſhot kind, worthy of remark as ſhould come within my obſervation; ſuch, I preſume, muſt alſo have been the determination of many of the medical men employed on this ſervice; and, moſt probably, ere now, you may have from this quarter, communications before you: ſtill, however, ſhould room be found, even in a ſecondary way, for theſe, I ſhall feel my purpoſe completely fulfilled.

I am, GENTLEMEN,

Your moſt obedient and humble ſervant,

*Schagen, North Holland,* J. CHRISTIE, 27th Foot.

*October* 16, 1799.

---

Before entering on my plan, I muſt firſt beg your indulgence to introduce here a few prefatory obſervations, not unconnected with the ſubject, together with remarks on gun-ſhot wounds in general; and although I am afraid theſe by ſome will be thought ſtrange and foreign, ſtill I conceive them, in ſome meaſure, neceſſary, for reaſons that may preſently appear. Firſt of all, I muſt not raiſe the hope, or expreſs the aſſurance, that what I may have to ſay on this ſubject, has not before been handled in a much abler manner by other pens: of this I am fully aware, although ſome of thoſe hints may have eſcaped writers, or have been thought by them of ſo little importance as to be unworthy of notice. Great allowances will, I am ſure, be made for the circumſtances of local ſituation; for here we are removed from the aſſiſtance of men or books—I have neither to conſult; it may therefore happen that, while I am buoying myſelf up with the thought of ſome of my remarks being *original,* the public eye may regard them only as hints borrowed, at the ſame time that their importance is but little. In meeting this laſt opinion, however true, I would here beg leave to reply, that in very many ſituations in which man muſt act his part, too little attention has frequently frequently been paid to matters confidered fmall, and the expreffion—"Take care of fmall things, the great will take care of themfelves," is neither without it's meaning or importance. What one man does, I maintain it, another may. The flighteft actions, or even expreffions of an individual, have frequently marked his true character, as certainly as if all his motions had been obferved, and all his thoughts been revealed from his cradle to his manhood:—to fpeak plainly, the furgeon who applies a bandage clumfily, or ufes his lancet aukwardly, I fhould be by no means ready to come under the *cut* of his great knife.

Hoping, as I confidently do, that allowances will be made for any inaccuracy or deficiency that may happen in confequence of local fituation, (and few are the leifure hours) I muft likewife not forget to intreat confideration on account of perfonal circumftances for the regimental practitioner. It muft be obvious, that he has but few advantages, and but little opportunity for obfervation; fince, as you well know, on all fervices, the worft cafes are intrufted to the ftaff of the hofpital department; and as a great majority of the wounded here have been fent to the different military hofpitals in England, my views will be in fome meafure retarded, fince thofe were principally to obferve the progrefs and termination of hurts in all their varieties, and through all their ftages. It is, however, principally the trammels of a fubordinate ftation, that I have moft to regret, in preventing thofe remarks from being fo ufeful as could be wifhed.

In barbarous times, among favage nations, it had been cuftomary for the combatants in war to poifon their weapons of deftruction; fuch has been faid to have been practifed in later times, and among people dignifying themfelves with the name of civilized and polifhed. It was from this fource, no doubt, that the idea of the nature and treatment of gun-fhot wounds being myfterious, had firft arifen; and even ftill, among ourfelves, I cannot help remarking it, a remnant of that pedantic fort of importance, nourifhed by narrownefs of foul, may be feen occafionally wielding her dark implements: but as the reign of fuperftition draws to a clofe, that of reafon hath generally fucceeded, and gun-fhot wounds have been long known, at leaft in Europe, to require no other treatment but that of contufed and lacerated injuries. In fome refpects, however, they are certainly different as to their mode of infliction; the courfe a mufquet-ball fometimes takes, is fo remarkable as to defcribe almoft the half circle of the body, fometimes winding, fometimes even retrograde; retrograde; and this singularity hath been long ago observed by army surgeons, and even noticed by that favourite of nature and ornament of art, Mr. JOHN HUNTER.

The turning course, which a musquet-ball frequently takes on entering the body, may be accounted for by a fact well known to the artillery-men and *sharp-shooters*.* A cannon or musquet-ball, in its passage through the air, hitting some firm resisting body, shall be seen to move directly out of it's straight course, and to take one laterally, or ascendingly. By way of illustration, let us suppose the head of a pin fixed on a billiard-table, and struck by the moving ball centrically, its straight course would be altered to an ascending one; if the ball strikes more or less laterally, so will its course be altered more or less oppositely. Thus it is with cannon and musquet-shot; for, during the action which happened upon our landing, the 27th of August, on that bloody shore bordering the Texel, the sand hills afforded a sort of covering for the combatants; a convenient retreat for the medical people to dress their wounded; yet, still, behind those protecting eminences, I was surprised to find an accident or two from shot, at the very instant of dressing. I looked to my right, there was the sea, from which a gun-boat or two only plyed well their carronades; on my left was a plain, on which the enemy's cavalry were placed to watch our motions; from neither of those quarters could the balls have proceeded. I now observed several men wounded by the enemy in front, although placed in a situation where no shots could have reached us, but by this turning sort of course I have attempted to describe. Now, a ball striking the head, a rib, or any other bone, and glancing along them, as it frequently does, without any material injury, must undoubtedly be owing to its meeting laterally the resisting body, by which its force will be but partially impeded. Thus I have been sometimes surprised to find in gun-shot wounds in the extremities, that the bone remained sound, where the ball had *seemingly* entered directly upon it, passing over it.—It is therefore probable,

* These last are light armed infantry, generally using rifled pieces, and dressed in green cloathing. They are detached among woods and on road sides, for the purpose of scouring and preventing surprise; they are called by the Dutch and Germans, Jägers, and Grün Jägers; for steadiness and precision they are said to be vastly superior to other troops; and our continental friends, as well as enemies, seem to employ more of those riflemen than the English. This is by no means fair; for these fellows, skulking in woods and thickets, like serpents, prepare and send their deadly messengers on many an open and unwary soldier with dreadful exactness.

bable, that in ſhots from muſquets, the bone is ſeldom fractured, unleſs the ball ſtrikes centrically. Hard and firm bodies frequently alter the courſe of balls, ſo that death or injury is ſometimes prevented; we alſo know that elaſtic ſubſtances, particularly when lubricated, poſſeſs this quality in a very eminent degree. It ſeems extremely likely, in many of thoſe lucky ſhots which had penetrated the cavaties of the thorax or abdomen, miſchief had been prevented by the yielding quality of the lungs or inteſtines; and thus, in ſome way, the courſe of the ball changed, and prevented from entering their ſubſtance, analogous to packed wool or hair, &c. which are known to reſiſt powerfully the entrance of penetrating round bodies. When we reflect for a moment on the maternal care by which Nature has ſo admirably provided againſt accidents to important parts; the ſerpentine courſe the carotids aſſume on viſiting the moſt wonderfully intricate and eſſential part of all animal machines; and, indeed, the provident manner in which the whole arterial ſyſtem is diſtributed, and the thouſand other accommodations ſhe univerſally provides for the preſervation of the animal: it is not at all an improbable conjecture, that the omentum may be placed on the inteſtines, partly for the defence we have been ſpeaking of, ſince in moſt animals it became indiſpenſably neceſſary, that the abdomen ſhould have fleſhy walls.

Although experience may have taught, that the lungs and abdominal viſcera may be penetrated by bullets, and followed by recovery, I believe the well authenticated caſes on record are but few, and that many of thoſe at leaſt, which had paſſed in from one ſide, ſo as to come out at the other, had left the viſcera unhurt; for it is to be obſerved in gun-ſhot wounds, that there muſt be always a conſiderable deſtruction of the ſurrounding parts, unavoidably ariſing from laceration; and thus, the ſloughing muſt be both certain and extenſive.—The velocity with which balls enter, may tend, in wounds of the thorax and abdomen, to prevent miſchief by little or no opportunity being given for the entrance of the external air—the wound cloſing like a valve—the conſequent coagulable effuſion and inflammation continuing in a great meaſure afterwards to ſhut up the orifice.

An officer of rank in the army, while ſerving in the Weſt Indies in 1796, received a muſquet ball, which lamed his hand; and almoſt immediately afterwards, another entered a little to the right of the left breaſt, i. e. between it and the ſternum, the ball carrying along with it a piece of ſhirt and flannel waiſtcoat; it came out almoſt oppoſite, but but more outwardly, i. e. about two hand breadths from the ſpine.—The conſequence of this wound was great oppreſſion, difficulty of breathing, and occaſional bloody ſpitting. Both orifices continued long to diſcharge, and he returning to England ſoon after, apparently in a ſtate of conſumption, was deſired by the firſt medical advice in the kingdom, to adhere to a proper diet, chiefly conſiſting of milk and vegetables, and to truſt the cure to Nature.—There was not any emphyſematous appearance; but after twelve months languiſhing with diſtreſſing pain, cough, and dyſpnœa, he was reduced to a mere ſkeleton—his ſpirits fled—he became peeviſh—refuſed nouriſhment, and was given up for loſt; until an extraordinary accident, which, if it were not well authenticated, I ſhould hardly be ready to communicate. The houſe in which he reſided having been extraordinarily illuminated, on account of the victory gained by Lord Duncan over the Dutch in 1797, and the room in which he ſlept being in an high ſituation of the houſe, the ſmoke of the candles brought on ſuch an intolerable fit of coughing, that a great quantity of frothy, bloody matter (or as he himſelf expreſſed it, all kinds of devils) came up, which had nearly ſuffocated him. Although his room was almoſt immediately changed, he remained the whole night, and for ſeveral days afterwards, in the greateſt diſtreſs from the cough and dyſpnœa.—After this, however, he gradually recovered—his cough left him, and with it all expectoration—he regained his appetite and ſtrength—one wound entirely healed—the other continued to diſcharge, and occaſionally to exfoliate a little bone. Twelve months after the accident, it diſcharged a hard ſubſtance, which was afterwards found to be a bit of flannel waiſtcoat, encruſted with an earthy kind of matter.—After this, he perfectly recovered, with only a ſlight hollowneſs of voice, and occaſional pain in the ſide, on any extraordinary exertion. From the imperfection in the voice, it is probable, the ſubſtance of the lungs had been penetrated. The ſame officer, in the action on the coaſt of Holland, on the 27th of Auguſt, received a muſquet ſhot, which glanced along the ſquamous portion of the temporal bone, carried away a portion of the orbit, and entirely deſtroyed the left eye; and although he is thus, as it were, broken winded, blind, and lame from wounds, he is yet healthy, active, vigorous, and in good ſpirits!

When ſpeaking of the eye, I would here beg to mention, that very obſtinate ophthalmies were frequently occaſioned on the coaſt of Holland, by the ſand blowing between and within the eye-lids.—This ſand

ſand was ſo fine and penetrating, that I believe it inſinuated itſelf into *every* aperture of the human body: I have obſerved in thoſe, as well as other ophthalmies, that reſiſt for a time the antiphlogiſtic plan, as bleeding, ſaturnine applications, &c. another ſort of treatment ſoon becomes neceſſary—the diviſion of the turgid veſſels on the conjunctiva, by the edge of a lancet, as mentioned by Dr. CULLEN, I have conſtantly found, *at firſt,* to afford conſiderable temporary relief, as well as leeching and cupping. Bliſtering is almoſt always very ſerviceable, but principally after the diſeaſe has been of ſome ſtanding, and then it appears to be uſeful by its ſtimulating quality; for the veſſels, after having been long turgid, become languid and inactive; and being principally connected to an unyeilding ſubſtance, they loſe the power of leſſening their diameters to their original ſize.

In obſtinate ophthalmies, I have frequently admired the care which Nature has ſeemingly taken to prevent ſuppuration; for here its formation is both leſs frequent, and leſs extenſive, than we would à priori expect; and it appears to me, by no means unlikely, that the blood, on entering the coats of the eye and its appendages, may undergo ſuch a change, as to render it leſs fit for the formation of pus; it may poſſibly part with its coagulable lymph; and, from the aſtoniſhing ſecretion that ſometimes happens in the lachrymal glands of children and hyſterical women, we would be led to imagine, that there was an exceſs of the watery part in theſe veſſels.

[To be continued in our next Number.]

